# Energy Homeostasis Disruption in Neurological Disorders: Mitochondrial Dysfunction, High-Energy Phosphate Transfer, and Extracellular ATP-Dependent Purinergic Dysregulation

**DOI:** 10.3390/ijms27136066

**Published:** 2026-07-06

**Authors:** Hirotaka Tao, Koichi Fujisawa

**Affiliations:** Department of Environmental Oncology, Institute of Industrial Ecological Sciences, University of Occupational and Environmental Health, Japan, 1-1 Iseigaoka, Yahatanishi-ku, Kitakyushu 807-8555, Fukuoka, Japan; fujisawa@med.uoeh-u.ac.jp

**Keywords:** energy homeostasis, mitochondrial dysfunction, high-energy phosphate transfer, extracellular ATP-dependent purinergic dysregulation, glia-dependent inflammation

## Abstract

Mitochondrial dysfunction and impairment of high-energy phosphate transfer are increasingly recognised as shared pathogenic features across neurological disorders. Because neurons require large amounts of ATP to sustain synaptic transmission, ion gradients, axonal transport, and intracellular signalling, they are especially vulnerable to disturbances in energy metabolism. Neurological dysfunction, therefore, cannot be explained solely by reduced mitochondrial ATP production. It also involves failure of the creatine kinase/phosphocreatine (CK/PCr) and adenylate kinase/AMP-activated protein kinase (AK–AMPK) systems, which normally support local ATP buffering, high-energy phosphate transfer, and intracellular energy homeostasis. In parallel, extracellular ATP-dependent purinergic dysregulation contributes to glia-mediated inflammation, synaptic dysfunction, and cell death, linking intracellular energy failure to abnormal intercellular signalling. In this review, we integrate these mechanisms into a shared pathological continuum of disrupted energy homeostasis. We then compare Alzheimer’s disease, Parkinson’s disease, and epilepsy as representative disorders with shared and disease-specific manifestations of this continuum, characterised respectively by chronic cerebral energy crisis, selective metabolic fragility, and acute energy overload with purinergic dysregulation. Finally, we discuss how this comparative perspective may help identify shared therapeutic opportunities while preserving disorder-specific interpretation.

## 1. Introduction

Mitochondrial dysfunction is increasingly recognised as a shared pathological feature across a wide range of neurological disorders, including Parkinson’s disease, Alzheimer’s disease, Huntington’s disease, and mitochondrial encephalopathies [1,2,3,4]. In these conditions, reduced electron transport chain activity, impaired mitochondrial quality control, excessive reactive oxygen species production, and disturbed calcium homeostasis converge to compromise cellular energy metabolism [1,5,6,7]. As a result, synaptic dysfunction, abnormal neurotransmitter release, and activation of cell death pathways may occur [8,9,10,11]. Maintenance of mitochondrial integrity and ATP-producing capacity is therefore essential for preserving neuronal function and intracellular energy homeostasis [1,2,12,13].

Neurons require large amounts of ATP to sustain ion gradients, synaptic transmission, axonal transport, and intracellular signalling, and are therefore especially vulnerable to disturbances in energy metabolism [2,12,14,15]. However, neurological dysfunction cannot be explained solely by reduced mitochondrial ATP production. It also involves impairment of high-energy phosphate buffering and transfer through the creatine kinase (CK) and adenylate kinase (AK) systems, which can amplify local energy insufficiency and metabolic vulnerability [16,17,18,19]. Accordingly, disruption of energy homeostasis in neurological disorders may be viewed not simply as a metabolic defect but as a shared pathological process linking mitochondrial dysfunction, impaired high-energy phosphate transfer, and extracellular ATP-dependent purinergic dysregulation.

**Figure 1:**
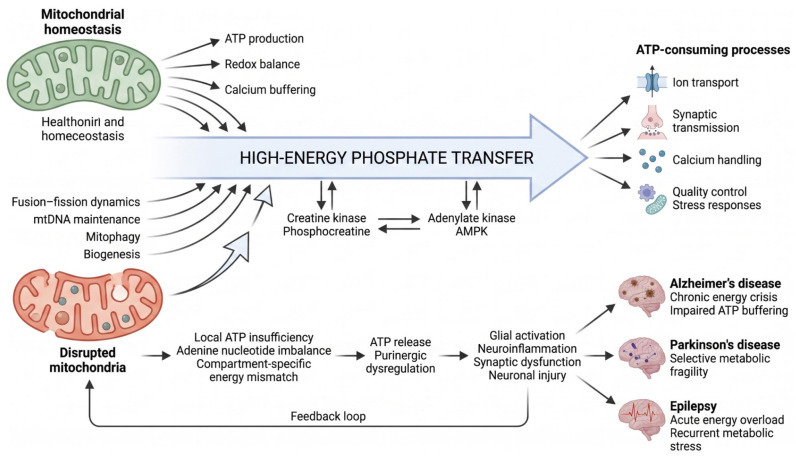
Integrated framework of mitochondrial homeostasis and energy homeostasis disruption in neurological disorders. Mitochondrial homeostasis supports neuronal and glial energy balance through ATP production, redox regulation, calcium handling, mitochondrial dynamics, mitophagy, and biogenesis. High-energy phosphate transfer, mediated by the creatine kinase/phosphocreatine and adenylate kinase/AMPK systems, links mitochondrial ATP generation to ATP buffering and local energy distribution. Disruption of mitochondrial homeostasis impairs these phosphotransfer systems, leading to local ATP insufficiency, metabolic inflexibility, purinergic dysregulation, neuroinflammation, synaptic dysfunction, and neuronal injury. This framework highlights Alzheimer’s disease, Parkinson’s disease, and epilepsy as disease-specific manifestations of impaired mitochondrial energy generation and high-energy phosphate transfer. Created in BioRender. Tao, H. (2026) https://BioRender.com/e16hw2c, accessed on 10 June 2026.

From this perspective, the present review reinterprets disruption of energy homeostasis in neurological disorders as a shared pathological continuum linking mitochondrial dysfunction, failure of high-energy phosphate transfer, and extracellular ATP-dependent purinergic dysregulation (Figure 1). We first outline the general principles of this framework and then examine the CK/PCr and AK-AMPK systems, together with extracellular ATP regulation, as interconnected mechanisms of metabolic vulnerability. We next compare Alzheimer’s disease, Parkinson’s disease, and epilepsy as representative disorders showing shared and disease-specific manifestations of this continuum. Finally, we discuss how this comparative perspective may help identify shared therapeutic opportunities while preserving disorder-specific interpretation.

## 2. General Overview of Energy Homeostasis Disruption in Neurological Disorders

### 2.1. Concept of Energy Homeostasis and Distinctive Features of the Nervous System

Energy homeostasis refers to the ability of cells to balance ATP production and consumption in response to changing demand and supply, thereby preserving stable function [12,13,20]. The brain is especially energy-demanding relative to its mass but has only limited intrinsic energy reserves. It therefore depends on tightly coordinated regulation of cerebral blood flow, substrate delivery, and intercellular metabolic coupling. Because neuronal energy demand changes dynamically with neural activity, neurometabolic coupling is a fundamental mechanism for maintaining homeostasis [12,14,21]. Systemic regulatory processes associated with sleep–wake state, ageing, and inflammation also influence this balance. For this reason, energy homeostasis provides a useful framework for understanding neurological disease [22,23,24].

Energy homeostasis may be viewed not as a fixed state but as a dynamic system that is continuously reconfigured by neuronal activity, sleep–wake state, ageing, and inflammatory conditions. Accordingly, metabolic abnormalities in neurological disorders are better understood as failures of coordination among neurons, astrocytes, oligodendrocytes, microglia, and the vascular system than as isolated pathway defects. This perspective also highlights metabolic alterations that emerge before overt neurological symptoms and supports the rationale for early diagnosis and preventive intervention [25,26,27].

### 2.2. Fundamentals of Energy Metabolism in the Central Nervous System

Energy metabolism in the central nervous system depends primarily on glucose, which is processed through glycolysis, the tricarboxylic acid (TCA) cycle, and oxidative phosphorylation to generate ATP. Neuronal activity requires a continuous ATP supply for membrane repolarisation, synaptic vesicle cycling, and neurotransmitter reuptake, making the brain highly metabolically active even at rest. At the same time, the brain can also use lactate, glycogen-derived substrates, and ketone bodies depending on physiological and pathological conditions. Metabolic dysfunction in neurological disorders should therefore be understood not simply as ATP deficiency but also as impaired metabolic flexibility and disrupted substrate utilisation [12,13,14,21].

Because neuronal activity can increase energy demand within seconds, substrate uptake, pathway switching, and local blood flow must be tightly coordinated. Although glucose is the principal substrate, lactate can also serve as a neuronal fuel and signalling molecule, and ketone body utilisation increases during fasting, development, and some therapeutic conditions [21,28,29]. This flexibility supports neural activity under physiological conditions, but may be progressively compromised under pathological conditions. Ageing, vascular dysfunction, inflammation, and declining mitochondrial function all reduce the brain’s ability to switch efficiently between substrates and adapt to fluctuating demand.

Understanding neurological disorders therefore requires attention not only to individual metabolic pathways but also to the progressive loss of metabolic reserve and adaptability in the brain [13,25,30,31].

### 2.3. Metabolic Interactions Among Neurons, Astrocytes, Oligodendrocytes, and Microglia

Brain energy metabolism depends on coordinated interactions among neurons, astrocytes, oligodendrocytes, and microglia. Neurons rely heavily on oxidative phosphorylation, whereas astrocytes are relatively more glycolytic and help support neuronal activity by supplying lactate and antioxidant metabolites. Oligodendrocytes contribute metabolic support to axons and help sustain long-range conduction in white matter. Although microglia are often grouped with glial cells in discussions of CNS function, they are ontogenetically distinct immune cells of myeloid/macrophage lineage rather than neuroectoderm-derived cells like neurons and astrocytes. Neuron–astrocyte–oligodendrocyte metabolic coupling, together with microglial immunometabolic responses, is therefore essential for both normal neural function and disease progression [21,31,32,33].

Microglia should also be viewed as metabolically responsive cells that change functional state according to energy conditions. A shift towards glycolysis in activated microglia is closely associated with pro-inflammatory activity and can increase metabolic stress on surrounding neurons under chronic conditions. Astrocytes, oligodendrocytes, and microglia should therefore be regarded not simply as support elements but as active determinants of whether energy homeostasis is maintained or disrupted in neurological disease [25,34,35,36].

### 2.4. Molecular Mechanisms Regulating Energy Homeostasis

Energy homeostasis in the nervous system is maintained by metabolic sensors and transcriptional regulators, including AMPK, mTOR, SIRT1, and PGC-1α. AMPK responds to energy depletion by promoting catabolic pathways, autophagy, and mitochondrial preservation, and together with SIRT1 and PGC-1α supports mitochondrial biogenesis and metabolic adaptation [37,38,39,40]. By contrast, mTOR regulates cell growth, protein synthesis, and nutrient-responsive anabolic signalling, and both excessive activation and suppression can be detrimental to neuronal function [41,42,43,44]. Energy homeostasis may therefore be understood not as a simple on–off system but as a dynamic balance among multiple metabolic regulators operating across time and cellular context.

### 2.5. Major Mechanisms of Energy Homeostasis Disruption in Neurological Disorders

Major mechanisms underlying disruption of energy homeostasis in neurological disorders include mitochondrial respiratory chain dysfunction, oxidative stress, impaired calcium homeostasis, breakdown of blood–brain barrier and dysregulation of cerebral blood flow, glia-dependent inflammation, and failure of intercellular metabolic coupling. Among these, mitochondria are especially important because they regulate not only ATP production but also reactive oxygen species control, calcium buffering, apoptosis, and proteostasis. Once mitochondrial function is compromised, multilayered cellular failure can follow. Abnormal protein accumulation can further aggravate energy metabolism by impairing mitochondrial transport, reducing membrane potential, and disrupting autophagy, whereas energy deficiency in turn weakens protein quality control. These processes therefore reinforce one another and help drive disease progression [1,45,46,47].

### 2.6. Mitochondrial Dysfunction, Neuronal Impairment, and Neurodegeneration

Taken together, the evidence reviewed above positions mitochondrial dysfunction as a shared pathological mechanism across neurodegenerative disorders [2,3,45,46]. Neurons are especially vulnerable because they depend on mitochondria to sustain synaptic transmission, axonal transport, ion gradients, and calcium homeostasis [2,4,14,15]. Even relatively early abnormalities—such as reduced ATP production, excessive reactive oxygen species (ROS) generation, loss of membrane potential, and defective calcium handling—can accumulate into progressive functional deficits [1,2,46]. This is particularly important in neurons with long axons, which require continuous energy delivery to distal regions. These structural and functional features provide an important background for understanding selective neuronal vulnerability in neurodegenerative disease [2,3,46].

Disease progression in neurodegenerative disorders is influenced not only by energy metabolic failure itself but also by disruption of mitochondrial quality control, including mitophagy, mitochondrial dynamics, and cell death regulation [1,2,6,45,46,48]. Imbalance in mitochondrial fusion and fission, impaired mitophagy, reduced mitochondrial biogenesis, and loss of mitochondrial DNA homeostasis all promote accumulation of dysfunctional organelles and persistent oxidative stress [1,6,45,46,47,48]. In addition, mitochondria-derived mtDNA and oxidised lipids can act as danger-associated molecular patterns, thereby inducing microglial activation and chronic glia-dependent inflammation [35,36,49]. This inflammatory response may further exacerbate mitochondrial injury, protein aggregation, synaptic dysfunction, and activation of cell death pathways such as apoptosis and ferroptosis [3,11,45,49,50]. Thus, mitochondrial dysfunction may be viewed not merely as a consequence of neurodegeneration but as a major pathological mechanism that can autonomously drive disease progression [2,3,45,46].

Mitochondrial dysfunction also interacts with disease-specific abnormal proteins and genetic backgrounds, including Aβ and tau in Alzheimer’s disease, α-synuclein in Parkinson’s disease, SOD1 and TDP-43 in amyotrophic lateral sclerosis, and mutant huntingtin in Huntington’s disease, thereby amplifying progression [3,4,45,46,47,51]. Disease-specific factors impair mitochondrial function, while dysfunctional mitochondria in turn promote protein aggregation, intracellular homeostatic failure, and neural circuit dysfunction. These processes therefore form a self-reinforcing pathological cycle. From this perspective, mitochondrial dysfunction is not only a common vulnerability across neurodegenerative diseases but also a molecular platform that amplifies disease-specific pathology [2,3,4,45,46].

When mitochondrial dysfunction causes energy failure that can no longer meet neuronal demand, functional abnormalities tend to appear before overt structural degeneration. Early changes include reduced synaptic efficiency, impaired maintenance of membrane potential, and defective axonal transport. If these disturbances persist, ATP depletion, reactive oxygen species, and calcium overload can activate excitotoxic and cell death pathways. In parallel, local metabolic failure promotes glial reactivity and inflammatory cytokine production, spreading secondary stress to surrounding tissue. Mitochondrial dysfunction may therefore be regarded not simply as a metabolic defect but as a central mechanism linking neural circuit failure to neurodegeneration [2,3,45,46].

The effects of mitochondrial dysfunction extend beyond molecular abnormalities within neurons and emerge as impaired information processing at the neural circuit level. Presynaptic vesicle refilling, neurotransmitter release, postsynaptic receptor function, and local protein translation may all be affected. As a result, higher-order functions such as memory, motor control, and sensory processing can be compromised. This is especially relevant in projection neurons with long axons, in which defective mitochondrial transport and local ATP insufficiency may serve as initiating factors for axonal degeneration and selective vulnerability [1,2,3,15,52].

Because chronic mitochondrial dysfunction can produce reversible functional decline before irreversible cell loss occurs, identifying metabolic vulnerability at this stage is especially important. To understand its molecular basis, attention must also be directed to the buffering and transfer systems that handle high-energy phosphate generated by mitochondria. In the next section, we therefore turn to the CK/PCr and AK systems and discuss the physiological and pathological significance of high-energy phosphate transfer in neurological disorders.

## 3. Failure of High-Energy Phosphate Transfer Systems

**Figure 2:**
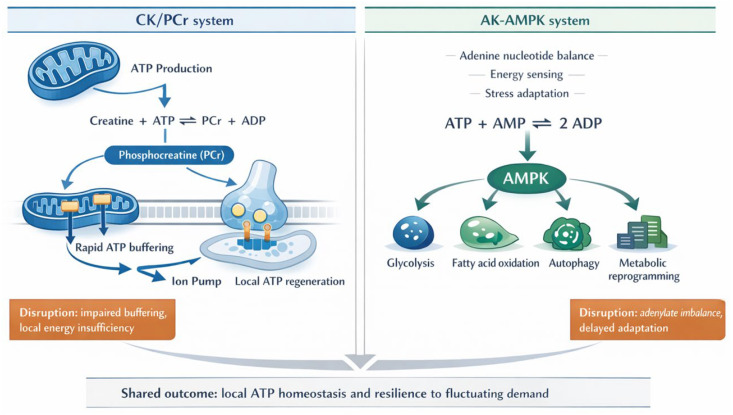
Complementary roles of the CK/PCr and AK-AMPK systems in high-energy phosphate transfer. The CK/PCr and AK-AMPK systems maintain intracellular energy homeostasis through complementary mechanisms. The CK/PCr system functions mainly as a local ATP buffering and spatial transfer system, delivering high-energy phosphate from mitochondria to sites of ATP consumption via phosphocreatine (PCr) and enabling rapid ATP regeneration. By contrast, the AK system preserves adenine nucleotide balance through the reaction ATP + AMP ⇄ 2 ADP and links nucleotide fluctuations to AMPK-dependent energy sensing and adaptive metabolic reprogramming. Through this pathway, the AK-AMPK axis supports glycolysis, fatty acid oxidation, autophagy, and other stress-responsive metabolic adjustments. Failure of the CK/PCr system impairs local ATP buffering and high-energy phosphate transfer, whereas AK dysfunction promotes adenylate imbalance and reduced adaptive capacity. Together, these systems sustain intracellular energy homeostasis and resilience to fluctuating metabolic demand. Created in BioRender. Tao, H. (2026) https://BioRender.com/hxvaxn2, accessed on 10 June 2026.

As outlined in the previous section, disruption of energy homeostasis cannot be explained solely by reduced ATP production. Impaired high-energy phosphate transfer and local ATP buffering at sites of demand may amplify metabolic failure. In neurons, in particular, the CK/PCr and AK-AMPK systems serve as major complementary mechanisms that help maintain intracellular energy homeostasis. In this section, we discuss the physiological significance of these high-energy phosphate transfer systems and the pathological implications of their disruption in neurological disorders (Figure 2).

Although both the CK/PCr and AK systems participate in high-energy phosphate transfer, their pathological implications are not identical. The CK/PCr system is primarily understood as a rapid buffering and transport mechanism that responds to local ATP demand, whereas the AK system regulates adenine nucleotide balance and can also connect to AMPK-dependent metabolic reprogramming and stress responses. In this review, we discuss the AK system in greater detail because it serves as a bridge between energy buffering and metabolic signalling, a feature that is particularly relevant for understanding neurological disease [16,17,18,19,53,54].

The CK/PCr system is a central high-energy phosphate transfer mechanism that supports intracellular energy homeostasis [18,19,49]. CK catalyses the reversible transfer of a phosphate group between ATP and creatine, and PCr functions as a storage form of high-energy phosphate [18,19,49]. CK isoforms include CK-MM, CK-MB, and CK-BB, and their tissue-specific distribution has long been used to infer organ-specific injury and neuromuscular involvement [48,50,51].

ATP generated in mitochondria is converted to PCr by mitochondrial CK. PCr then diffuses through the cytoplasm and is used to regenerate ATP at sites of high demand, such as synaptic terminals and ion pumps [18,49,55,56]. The CK/PCr system therefore functions both as a temporal buffer that dampens abrupt fluctuations in ATP concentration and as a spatial energy transport system that delivers high-energy phosphate from mitochondria to ATP-consuming compartments [18,49,52,56]. In neurons, this mechanism is particularly important for meeting rapidly changing local energy demand during neural activity. Under conditions of mitochondrial dysfunction, reduced ATP supply, abnormal mitochondrial localisation, and altered CK isoform activity can together increase the likelihood of local energy insufficiency [18,55,57,58].

More specifically, mitochondrial CK is functionally coupled to oxidative phosphorylation near the outer mitochondrial membrane and contact sites involving voltage-dependent anion channels and adenine nucleotide translocase, where newly generated ATP is rapidly used to phosphorylate creatine [18,49,52,55,56]. The resulting PCr has a smaller effective diffusion barrier than ATP–ADP exchange in crowded cytoplasm and can therefore carry high-energy phosphate away from mitochondria toward spatially restricted ATP-consuming domains [18,49,55,56,59,60,61]. At presynaptic terminals, this local ATP regeneration is relevant because synaptic vesicle endocytosis and recycling, vesicular neurotransmitter loading, exocytotic release, and Ca^2+^ clearance after firing all impose abrupt and compartmentalised ATP demand [14,60,62]. Along the plasma membrane and axon, PCr-dependent ATP regeneration can help sustain Na^+^/K^+^-ATPase activity, membrane repolarisation, cytoskeletal remodelling, and fast axonal transport, processes that are especially vulnerable in long projection neurons where mitochondria cannot always be positioned immediately adjacent to every energy-consuming site [3,15,52,60,61,63]. In this sense, the CK/PCr system should be viewed not only as a passive energy reservoir but also as a phosphocreatine shuttle that links mitochondrial ATP production to synapses, ion pumps, transport motors, and distal axonal compartments [18,49,52,55,56,59,60,61]. When mitochondrial positioning, creatine availability, PCr diffusion, or CK-BB activity is impaired, ATP may remain relatively preserved at the whole-cell level while local ATP-consuming microdomains become energy insufficient; this compartment-specific mismatch provides a mechanistic explanation for early synaptic failure, impaired excitability control, and axonal vulnerability in neurological disorders [49,56,58,60,61,64].

### 3.1. Disruption of the CK System in Neurological Disorders

In the central nervous system, disruption of the CK system is thought to contribute to impaired energy homeostasis [49,64]. Brain-type CK-BB plays a major role in ATP regeneration and phosphocreatine buffering in neurons, thereby supporting sustained firing activity and synaptic transmission [49,55,56,58]. Accordingly, mitochondrial dysfunction or reduced CK activity may amplify neuronal injury by promoting local energy insufficiency [55,58,64]. In the brain, the CK/PCr system is also increasingly accessible to in vivo investigation. Advances in 31P magnetic resonance spectroscopy have enabled quantitative analysis of CK reaction rates and ATP synthesis dynamics in the human brain [49,65,66,67]. These findings suggest that the CK/PCr system is not confined to muscle energetics but also plays a critical role in maintaining energy homeostasis in the central nervous system. Moreover, altered expression or activity of brain-type CK-BB has been reported in neurodegenerative disorders, including Alzheimer’s disease, further supporting a role for CK/PCr system failure in neuronal dysfunction and disease progression [49,64].

Abnormalities in CK have been linked to the energy metabolic disturbances observed in disorders such as Alzheimer’s disease and Parkinson’s disease, where they may act together with oxidative stress, LRRK2-related pathways, and mitochondrial dysfunction to promote neurodegeneration [8,18,57,64]. In stroke and traumatic brain injury, abrupt energy imbalance also develops, and disruption of CK responses during recovery may contribute to secondary injury.

In epilepsy, where excessive neuronal activity sharply increases energy demand, the CK system is thought to contribute to short-term energy compensation. Indeed, changes in CK activity have been reported in experimental epilepsy models, suggesting that vulnerability of the CK/PCr system may aggravate energy failure during seizures [19,47,49,68].

Collectively, the significance of the CK/PCr system in the nervous system is supported by both human 31P-MRS studies and preclinical models [65,66,67,69,70,71]. However, it remains unclear whether changes in CK activity in individual neurological disorders are causative drivers of disease progression or secondary consequences of ongoing pathology. The CK/PCr system should therefore be regarded as an important component of metabolic vulnerability in neurological disease, while its causal contribution awaits further clarification.

### 3.2. The AK System and Adenine Nucleotide Homeostasis

Cells continuously adjust the balance between ATP production and consumption in order to sustain essential processes such as growth, differentiation, excitatory signalling, and stress responses. Maintenance of this balance depends not only on the absolute amount of ATP but also on precise control of the adenine nucleotide ratio among ATP, ADP, and AMP. Disruption of this balance can propagate to metabolic failure, cellular dysfunction, and tissue injury. The AK system is a central enzymatic network that maintains nucleotide balance through the reversible reaction ATP + AMP ⇄ 2 ADP. It therefore plays a broad role in sustaining intracellular energy homeostasis [16,17,53,54].

In humans, nine AK isoforms (AK1–AK9) have been identified and are distributed across the cytosol, mitochondria, and nucleus [16,17,72]. AK1, AK5, AK7, and AK8 are mainly cytosolic; AK2 is localised to the mitochondrial intermembrane space; AK3 and AK4 reside in the mitochondrial matrix; and AK6 and AK9 are nuclear. Each isoform is thought to perform distinct functions according to its subcellular localisation and tissue specificity [16,17,73,74].

AK is now understood not merely as a phosphate-transfer enzyme but as part of a broader molecular network that reflects a compartment-specific energy state and mediates metabolic signalling and stress adaptation. In particular, AMP generated by the AK reaction provides a key signal for activation of AMP-activated protein kinase (AMPK), thereby linking short-term energy buffering with downstream responses such as glycolysis, fatty acid oxidation, autophagy, and suppression of biosynthetic pathways. From this perspective, the AK system may be viewed as an integrative node that coordinates energy buffering, metabolic sensing, and signalling control [16,17,53,54].

Recent evidence has also clarified that individual AK isoforms differ in both physiological role and disease relevance according to their intracellular localisation. AK2 is associated with congenital immunodeficiency, AK7 with ciliary dysfunction, AK4 and AK6 with cancer metabolism, and AK5 with neurological disease [75,76,77,78,79,80,81,82,83,84,85,86,87,88]. Although these observations have steadily expanded the clinical significance of AK isoforms, it remains insufficiently understood how they divide labour within intracellular energy networks, particularly in high-energy-demand cells such as neurons.

The reversible reaction ATP + AMP ⇄ 2 ADP becomes particularly important under metabolic stress, when ATP consumption is increased. By generating ATP and AMP from two molecules of ADP, AK helps preserve adenine nucleotide balance and adenylate energy charge while limiting accumulation of ADP, which can otherwise inhibit ATP-dependent reactions [17,53,89].

Among AK isoforms, AK4 and AK5 have attracted particular attention in the nervous system. AK4 has been proposed to exert protective effects against ischemic stress: its expression decreases in models of focal cerebral ischemia and oxygen–glucose deprivation, whereas overexpression reduces infarct volume and cell death [16,90]. These effects are thought to involve preservation of ATP homeostasis, maintenance of mitochondrial function, and enhanced mitophagy through the PINK1/Parkin pathway [1,2,90].

Nevertheless, AK4 is increasingly regarded not as a constitutive housekeeping enzyme but as a mitochondria-related factor that functions according to cellular state and stress conditions. Early localisation studies reported cell-type-selective expression in mouse tissues, while typical AK activity was not clearly demonstrated [16,73]. More recent work in macrophages has shown that Ak4 contributes to mitochondrial DNA synthesis, maintenance of mitochondrial content, mtROS production, inflammatory signalling, and antibacterial defence, raising the possibility that AK4 may also function as a state-dependent immunometabolic regulator in other cell types [91].

Interpretation of AK4 enzymatic activity nevertheless still requires caution. Some early studies were negative regarding its catalytic activity, whereas more recent work supports condition-dependent activity. These discrepancies may reflect differences in cell type, experimental context, and assay systems. Reproducible validation under controlled experimental conditions will therefore be necessary to define the molecular function of AK4 more conclusively [16,73,90,91].

In addition, in the contexts of acute kidney injury and renal dysfunction associated with persistent heteroplasmic mtDNA mutations, AK4 has been suggested to contribute to ATP recovery following adenosine supplementation. This observation raises the possibility that AK4 may serve as a candidate determinant of responsiveness to nucleotide-corrective interventions [92].

AK5, originally characterised as a brain-associated isoform, is also expressed in the heart, pancreas, kidney, and intestine, where it may participate not only in metabolic regulation but also in differentiation and cellular responses [16,93,94]. In the nervous system, AK5 has been linked to autoimmune limbic encephalitis and epilepsy, suggesting a relationship to intracellular metabolic regulation and signalling [87,93,95,96].

AK5-deficient mice also exhibit reduced white adipose tissue mass during fasting, adipocyte shrinkage, altered lipid metabolism and inflammatory markers, decreased leptin signalling, and increased serum norepinephrine levels, suggesting possible involvement in brain–adipose tissue communication [97]. However, many of these findings remain at the preclinical stage, and caution is warranted when extrapolating them to human disease.

Taken together, the CK and AK systems function complementarily within intracellular energy networks despite handling different substrates. The CK system rapidly transfers high-energy phosphate through phosphocreatine to meet local ATP demand, whereas the AK system regulates ATP/ADP/AMP balance, preserves energy charge, and links nucleotide state to metabolic signalling [17,53,54,89]. In particular, AK-derived AMP can activate AMPK and thereby connect short-term energy compensation to longer-term stress adaptation and metabolic reprogramming [17,53,54]. Disruption of these systems therefore affects not only ATP availability but also broader signalling and survival pathways in neurons.

Although the AK–AMPK connection represents a biochemically and cell-biologically plausible framework, direct evidence demonstrating its causal contribution in human neurological disease remains limited. At present, therefore, the AK system should be regarded as a promising integrative candidate mechanism while its disease-modifying significance awaits further validation.

### 3.3. Molecular and Cellular Consequences of AK Dysfunction in Neurons

AK dysfunction can reduce neuronal energy reserve by disrupting adenine nucleotide balance and energy signalling, thereby exerting broad effects on synaptic function, membrane excitability, and cell survival [16,17,53].

When AK activity declines, ATP regeneration and ADP/AMP buffering are impaired, which may place a substantial burden on highly energy-dependent neuronal functions [16,17,53]. As a consequence, reduced neurotransmission efficiency, increased susceptibility to excitotoxicity, and induction of apoptotic or necrotic cell death pathways may occur [1,3,8,9].

AK and AMPK are functionally closely linked, and AMP generated through the AK reaction contributes to AMPK activation during energy stress [17,53,54,98]. AK dysfunction therefore represents more than simple ATP deficiency; it may increase cellular vulnerability by delaying metabolic reprogramming and stress adaptation [38,53,54,99]. This adaptive failure may manifest as insufficient induction of glycolysis, fatty acid oxidation, and autophagy, or as inadequate suppression of nonessential biosynthetic pathways [20,38,54]. As a result, recovery from metabolic stress is delayed and neurons may be exposed to sustained functional impairment and cumulative injury [38,39,54,99].

Furthermore, AK abnormalities may amplify energy imbalance across intracellular compartments [16,17,53]. In regions with high local demand, such as synapses, axons, and perimitochondrial domains, even modest disturbances in nucleotide balance may directly compromise neurotransmission and calcium homeostasis and thereby contribute to disease progression [1,2,10,17]. Such local vulnerability is likely to be intensified by differences in energy demand among the cytosol, mitochondria, and nucleus, and AMPK is one of the major factors coordinating metabolic reprogramming across these compartments [38,39,54]. The consequences of AK dysfunction should therefore be understood not only as failure of nucleotide balance but also as reduced stress tolerance due to insufficient AMPK-dependent metabolic adaptation [38,39,53,99].

To understand the AK–AMPK connection in neurons, it is therefore necessary to consider AMPK energy-sensing mechanisms and downstream metabolic responses in their own right. In the following section, we outline how AMPK detects nucleotide fluctuations and converts them into metabolic reprogramming.

### 3.4. Energy Sensing and Metabolic Reprogramming Through AMPK

AMPK is a central kinase that senses changes in the intracellular AMP/ADP/ATP ratio and reprograms metabolism from an energy-consuming state toward a recovery-oriented state under energy stress [38,98,100,101]. Once activated, AMPK suppresses ATP-consuming biosynthetic pathways while promoting glycolysis, fatty acid oxidation, autophagy, and other ATP-generating or ATP-sparing processes, thereby contributing to restoration of energy homeostasis [20,38,54,102].

AMPK is a heterotrimeric complex composed of a catalytic α subunit and regulatory β and γ subunits, each of which exists in multiple mammalian isoforms [100,103,104,105]. The β subunit contributes to complex stability, scaffold function, intracellular localisation, and substrate selectivity. The core activation mechanism involves AMP- or ADP-dependent promotion of Thr172 phosphorylation and inhibition of dephosphorylation. Upstream kinases include LKB1 and, under certain conditions, CaMKKβ and TAK1 [100,102,105,106]. In addition to changes in nucleotide ratio, AMPK can also respond to other metabolic inputs, such as aldolase-dependent sensing of glucose starvation and binding of long-chain fatty acyl esters. It is therefore best understood as a molecular platform that integrates diverse forms of metabolic stress [100,103,105,106]. Representative outputs include enhanced glucose utilisation: in skeletal muscle and ischemic myocardium, AMPK promotes GLUT4 translocation, while TXNIP phosphorylation and degradation increase GLUT1-dependent glucose uptake [98,101,102,106]. In the brain, AMPK is also thought to support adaptation to local energy insufficiency by maintaining astrocytic GLUT1 expression and increasing surface expression of neuronal GLUT3 [38,107,108,109].

Understanding cerebral glucose metabolism also requires consideration of the astrocyte–neuron lactate shuttle (ANLS). Glutamate release during neuronal activity induces Na^+^/K^+^-ATPase activation and enhanced glycolysis in astrocytes, and the resulting lactate is transported to neurons via monocarboxylate transporters [21,31,108,109]. In this process, AMPK helps support adaptation to local energy demand by maintaining astrocytic glucose uptake, activating glycolytic enzymes, and promoting lactate production. In particular, increased fructose-2,6-bisphosphate synthesis through 6-phosphofructo-2-kinase may contribute to enhanced glycolytic flux [38,103,108,109].

AMPK is involved not only in acute metabolic regulation but also in more sustained metabolic reprogramming through phosphorylation of transcription factors and coactivators [38,54,103,110]. Through these effects, cells can maintain energy conservation and substrate switching over the medium to long term. In the nervous system, such metabolic reprogramming is thought to connect to neuroprotection, neural circuit function, and even organism-level regulation of feeding and metabolism [38,110,111].

The effects of AMPK in the nervous system are not uniform; depending on neuronal subtype, the nature of the insult, and the intensity and duration of activation, AMPK may exert either neuroprotective or injury-promoting actions [38,99,108,110]. Indeed, protective effects have been reported in models of starvation, chemical ischemia, and excitotoxicity, whereas excessive or prolonged activation may also facilitate cell death [38,99,108,112]. AMPK should therefore be understood not as a simple protective factor but as a context-dependent regulator of metabolic stress responses.

AMPK has also been implicated in neurodevelopment and in maintenance of function in the mature brain [106,107,109]. Although some studies suggest that AMPK deletion during embryogenesis has relatively limited effects, excessive activation during critical developmental windows may influence cell polarisation, axonal extension, and dendritic formation [107,109]. In postnatal and ageing brains, AMPK deficiency has been associated with increased baseline excitability, heightened seizure susceptibility, and cortical atrophy linked to impaired neural stem cell maintenance, suggesting that appropriate AMPK activity is important for preserving neural circuit homeostasis [106,107,109].

AMPK is also thought to participate in neuronal responses to metabolic stress, excitotoxicity, and oxidative stress, and may support synaptic function and cell survival through receptor phosphorylation and preservation of mitochondrial function [38,39,107,113]. In addition, AMPK has been implicated in neuronal polarity, growth, and plasticity, suggesting that its role in the nervous system extends beyond acute metabolic compensation to long-term maintenance of neural circuitry [38,106,107,109].

Hypothalamic AMPK activity also regulates whole-body energy balance through feeding behaviour, thermogenesis, and sympathetic output. In general, fed state and hormones such as leptin, insulin, thyroid hormone, GLP-1, and oestradiol suppress hypothalamic AMPK activity, whereas fasting and ghrelin activate it [68,106,111,112]. These regulatory effects are reflected in brown adipose tissue thermogenesis and white adipose tissue browning, indicating that central AMPK functions not only in intracellular metabolic control but also in integrated organism-level energy regulation.

Together, AMPK functions intracellularly as a core regulator of metabolic reprogramming and, in the nervous system, as a factor connecting neuroprotection to organism-level energy homeostasis [38,103,110,111]. Distinguishing these dual roles is important for clarifying the significance of the AK–AMPK connection in neurological disease [17,53,54,110].

AMPK activity has also been linked to lifespan extension and attenuation of age-related functional decline in model organisms, and, in mammals, its activation by metabolic modulators or polyphenols may contribute to modification of ageing phenotypes [100,101,102,105,112,114]. However, because such effects may involve both AMPK-dependent and AMPK-independent mechanisms, interpretation of longevity-related actions in the nervous system still requires caution. Nevertheless, the view of AMPK as an integrator of energy sensing, substrate utilisation, neuroprotection, and organism-level metabolic regulation remains important for understanding neurometabolic disturbances and considering therapeutic intervention.

### 3.5. From High-Energy Phosphate Transfer to Extracellular ATP-Dependent Purinergic Dysregulation

The CK/PCr and AK-AMPK systems discussed above govern intracellular high-energy phosphate transfer and metabolic reprogramming, but their disruption may manifest not only as intracellular energy deficiency but also as quantitative and qualitative alterations in extracellular ATP-dependent purinergic dysregulation [22,23,103]. In differentiated C2C12 myotubes, for example, AK1 expression and extracellular ATP synthesis increase during differentiation, and this extracellular ATP production is abolished by AK1 knockdown but not by knockdown of ATP synthase β. Moreover, secretion of cytosolic AK1, rather than membrane-associated AK1β, is required for this process [115]. Although these findings were obtained in muscle cells and should not be directly extrapolated to the nervous system, they provide an important example indicating that AK may, under certain conditions, participate not only in intracellular adenylate balance but also in extracellular ATP production itself. Thus, fluctuations in intracellular adenine nucleotide balance, mitochondrial dysfunction, and local failure of high-energy phosphate transfer may secondarily alter ATP release, degradation, and receptor responses in neurons and glia, thereby amplifying inflammation and synaptic dysfunction [23,103,104].

**Figure 3:**
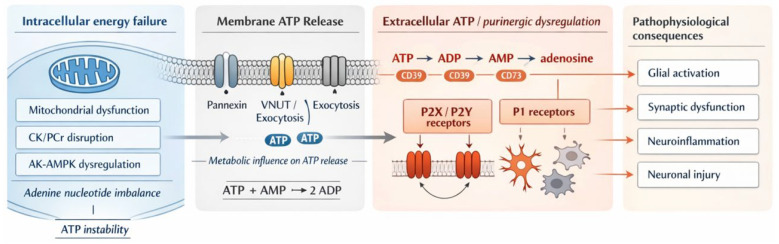
From intracellular energy failure to extracellular ATP-dependent purinergic dysregulation. This figure illustrates a mechanistic link between intracellular energy failure and abnormal intercellular signalling. On the intracellular side, mitochondrial dysfunction, together with failure of the creatine kinase/phosphocreatine (CK/PCr) system and dysregulation of the adenylate kinase/AMP-activated protein kinase (AK-AMPK) system, impairs local ATP buffering, adenine nucleotide balance, and adaptive metabolic responses, thereby promoting intracellular ATP instability. These intracellular abnormalities may influence ATP release across the plasma membrane through pathways such as pannexin channels, vesicular nucleotide transporter (VNUT)-dependent exocytosis, and volume-regulated anion channels (VRACs). Once released, extracellular ATP is sequentially converted to ADP, AMP, and adenosine by ectonucleotidases including CD39 and CD73, thereby shaping receptor signalling through P2X/P2Y and P1 receptors. Dysregulation of this extracellular purine environment contributes to glia-dependent inflammation, synaptic dysfunction, and neuronal injury. In this way, extracellular ATP-dependent purinergic dysregulation can be interpreted as a downstream expression of intracellular energy failure and a key interface linking metabolic stress to abnormal intercellular communication in neurological disorders. Created in BioRender. Tao, H. (2026) https://BioRender.com/c9cmuv7, accessed on 10 June 2026.

In neurological disorders, changes in extracellular ATP associated with intracellular energy failure may engage P2X/P2Y receptor-dependent inflammatory responses, regulation of synaptic transmission, and activation of cell death pathways, thereby forming a mechanistic bridge between intracellular metabolic dysfunction and abnormal intercellular signalling [7,23,40,104]. This relationship is summarised in Figure 3 as a transition from intracellular energy failure to extracellular ATP-dependent purinergic dysregulation. Understanding intracellular high-energy phosphate transfer systems is, therefore, a prerequisite for interpreting the pathophysiological significance of extracellular ATP-dependent purinergic dysregulation. In the next section, we turn to the basic framework of extracellular ATP release, reception, and degradation and discuss its relationship to inflammation, synaptic function, and cell death in neurological disease.

## 4. Extracellular ATP-Dependent Purinergic Dysregulation and the Regulation of Energy Metabolism

The high-energy phosphate transfer systems discussed in the previous section are not limited to maintaining intracellular metabolic homeostasis. They are also linked to intercellular communication through extracellular ATP release and purinergic signalling. In neurological disorders, metabolic failure may therefore be expressed not only as intracellular ATP deficiency but also as extracellular ATP-dependent purinergic dysregulation that affects inflammation, synaptic regulation, and cell death. In this section, we summarise the basic framework of eATP release, reception, and degradation and discuss its pathophysiological significance in neurological disease.

### 4.1. Basic Framework of Extracellular ATP Release, Reception, and Degradation

Extracellular ATP (eATP) is an important signalling molecule in the nervous system, where it exerts major effects on neuronal function, survival, and the inflammatory environment [7,12,20,36]. Its role is especially relevant in neurological disorders, in which injury, stress, and cell death can trigger large increases in eATP release. Once released, eATP activates P2X and P2Y receptors expressed on neurons, microglia, and astrocytes, thereby driving complex networks of cellular responses [7,20,27,36]. Depending on the context and receptor subtype involved, these responses may either aggravate neural injury or promote repair processes.

In addition to activating P2X and P2Y receptors, eATP is rapidly degraded by ectonucleotidases to ADP, AMP, and adenosine [22,116,117,118]. These metabolites have distinct receptor targets and physiological effects. Adenosine, for example, often exerts neuroprotective and anti-inflammatory actions through P1 receptors [22,114,118,119]. The extracellular environment therefore reflects a complex balance between excitatory and suppressive purinergic signals. Dysregulation of this enzymatic cascade may shift that balance toward either neurotoxicity or protection and thereby influence disease progression [22,116,117,118].

### 4.2. Feedback Regulation Between eATP Signalling and the AK/AMPK System

With regard to the relationship between extracellular ATP-dependent purinergic dysregulation and the AK/AMPK system, extracellular ATP has been reported to influence inflammatory responses and mitochondrial function through purinergic receptors, particularly the P2X7 receptor, and these processes are closely linked to intracellular metabolic state [120,121,122,123]. In differentiated C2C12 myotubes, secretion of cytosolic AK1 is required for the increase in extracellular ATP synthesis that accompanies differentiation. This observation supports the possibility that AK may contribute not only to intracellular nucleotide homeostasis but also to formation of the extracellular purine environment [115]. At the same time, only limited evidence directly demonstrates a bidirectional closed loop between eATP and AK. At present, it is therefore most appropriate to view extracellular ATP-dependent purinergic dysregulation and intracellular adenylate homeostasis as functionally interconnected systems rather than a fully established single regulatory loop [12,17,53,54].

Activation of purinergic receptors, particularly P2X7, has been linked to calcium influx, production of reactive oxygen species, altered mitochondrial respiration, and inflammasome activation. This pathway can therefore be regarded as a mechanistic interface linking inflammatory responses to changes in energy metabolism [120,121,122,124]. By contrast, AK maintains adenine nucleotide balance through the reaction ATP + AMP ⇄ 2 ADP and may also influence AMPK activation through changes in AMP concentration [17,53,54,98]. In addition, P2X7 stimulation has been associated with AMPK activation, autophagy, and mitochondrial quality control in certain immune cells and microglia. Taken as a whole, these findings suggest that the eATP/P2X7 system and the AK-AMPK system may functionally converge as a network supporting metabolic adaptation under inflammatory conditions [120,122,124,125].

Although individual findings concerning inflammation, mitochondrial function, and metabolic reprogramming support a functional connection between extracellular ATP-dependent purinergic dysregulation and the AK/AMPK system, few studies have demonstrated these processes as a single closed regulatory loop [120,121,122,123,124,125,126]. The framework proposed here should therefore be interpreted as incorporating both established evidence and hypothesis-driven extensions.

### 4.3. Effects on Synaptic Plasticity and Neurotransmission

Extracellular ATP can be released in the central nervous system as a neurotransmitter or cotransmitter and can modulate both presynaptic and postsynaptic function through P2X and P2Y receptors [62,124,127,128]. In particular, eATP may influence neurotransmitter release probability, receptor trafficking, calcium signalling, and the induction threshold for long-term potentiation and long-term depression. However, its effects may be either facilitatory or suppressive depending on receptor subtype, brain region, and stimulation conditions [128,129,130]. By contrast, AK redistributes high-energy phosphate through the reaction ATP + AMP ⇄ 2 ADP and can therefore be regarded as part of the intracellular machinery that adapts to local energy demand during neuronal activity [17,53,54].

Abnormalities in extracellular ATP-dependent purinergic dysregulation or adenosine metabolism have been linked to impaired synaptic transmission and disruption of neural circuit homeostasis in epilepsy, neurodegenerative disease, and ischaemic injury [12,39,40,130]. Excessive eATP or overactivation of P2X7 receptors has been reported to promote synaptic dysfunction and neurotoxicity through calcium influx, altered receptor function, and glia-dependent inflammation [119,126,130,131]. By comparison, direct evidence that reduced AK function causes synaptic dysfunction in the nervous system remains limited. Nevertheless, intracellular energy metabolic failure can impair essential processes required for neurotransmission, including vesicle recycling, maintenance of ion gradients, and axonal transport, and may thereby contribute to the breakdown of synaptic homeostasis [17,52,53,54,56].

### 4.4. Neuroinflammation

Neuroinflammation is a dual biological process that can support central nervous system homeostasis and tissue repair, but may also drive disease progression when excessive or sustained. Microglia-mediated neuroinflammation is a shared pathological feature of many neurodegenerative diseases, and its consequences depend on the balance between inflammatory and reparative responses. Although microglial activation has traditionally been described using M1-like and M2-like phenotypes, with M1-like states associated with inflammatory neurotoxicity and M2-like states linked to anti-inflammatory repair, this framework should be regarded as a simplified conceptual model [132]. In the acute phase, microglia may clear damaged cells, debris, and abnormal proteins and produce re-pair-associated mediators such as IL-10, TGF-β, and BDNF, thereby contributing to resolution of inflammation, tissue repair, and preservation of synaptic function [132].

When inflammatory stimulation becomes chronic, microglia may adopt a pro-inflammatory state characterised by IL-1β, IL-6, TNF-α, nitric oxide, reactive oxygen species, and prostaglandins. Excessive production of these mediators can disrupt the blood–brain barrier, impair synaptic transmission, induce mitochondrial dysfunction, increase oxidative stress, and promote neuronal death and neurodegenerative progression [60,132,133,134,135]. Ageing, trauma, and neurodegenerative disease can further prime microglia, leading to exaggerated responses to secondary stimuli and contributing to cognitive decline and progressive neurodegeneration [133,136]. Thus, neuroinflammation is reparative when appropriately regulated, but becomes neurotoxic when chronic and dysregulated.

Neuroinflammation is shaped by interactions among microglia, astrocytes, neurons, vascular endothelial cells, and peripheral immune cells. Cytokine-mediated increases in blood–brain barrier permeability can facilitate peripheral immune-cell entry, while reactive oxygen species promote lipid peroxidation, protein modification, DNA damage, and mitochondrial dysfunction [60,134,135]. Neuroinflammation should therefore be understood as a pathological network in which oxidative stress, blood–brain barrier dysfunction, metabolic disturbance, and synaptic impairment are mutually interconnected [60,134,135].

At the molecular level, the NLRP3 inflammasome is a key pathway regulating microglial inflammatory responses. Its activation promotes caspase-1-dependent maturation and release of IL-1β and IL-18, helping to establish chronic inflammation [61]. In neurodegenerative diseases, abnormal protein accumulation, mitochondrial dysfunction, and oxidative stress may trigger inflammasome activation and create a self-amplifying cycle of inflammation and neuronal injury [61].

However, the M1/M2 framework does not fully capture the diversity of microglial states in the central nervous system. Microglial phenotypes are dynamic continua shaped by disease stage, local microenvironment, anatomical region, and intercellular interactions [132]. Accordingly, therapeutic strategies should not suppress neuroinflammation indiscriminately, but should restrain neurotoxic pro-inflammatory responses while preserving reparative mechanisms involved in tissue repair and neuroprotection [132].

Single-cell RNA sequencing further demonstrates that microglia are heterogeneous and display transcriptional states that vary with development, ageing, disease context, and brain region [133]. In Alzheimer’s disease and related models, disease-associated microglia-like states show downregulation of homeostatic genes and upregulation of genes related to lipid metabolism, phagocytosis, and inflammation [133,134,137]. These findings emphasise that neuroinflammation should be interpreted through spatiotemporal and disease-specific changes in microglial function rather than by a fixed binary classification [132,133,134,137].

### 4.5. Significance of the eATP–AK Axis in Glia-Dependent Inflammation

Changes in the adenylate pool regulated by AK may influence AMP-dependent activation of AMPK and thereby represent a candidate pathway controlling metabolic responses during inflammation [17,53,54,109]. In many studies, AMPK activation suppresses NF-κB signalling and inflammasome activity and tends to reduce the production of inflammatory cytokines, although its effects also depend on cell type, stimulation conditions, and the degree of activation [109,120,121,138]. Glia-dependent inflammation may therefore involve interactions between eATP/P2X7-dependent inflammatory amplification and AK-AMPK-dependent metabolic adaptation and inflammatory control. However, the specific causal relationships and their disease-specific differences remain to be clarified.

Thus, the interaction between eATP–P2X7-dependent signalling and AK-AMPK-dependent metabolic responses provides a useful integrative perspective for understanding glia-dependent inflammation [120,121,122,123,124,138]. Importantly, these interactions are highly cell-type- and context-dependent, and caution is required when generalising them across human neurological diseases.

### 4.6. Extension to Rare Neurological and Neurodevelopmental Disorders

In addition to classical neurodegenerative and ischaemic disorders, extracellular ATP-dependent purinergic dysregulation and abnormalities in adenosine metabolism are also being investigated in certain neurodevelopmental conditions and rare neurological diseases [12,30,130,139]. Changes in P2 receptor signalling, extracellular ATP dynamics, adenosine turnover, and glia-mediated neuroimmune interactions have been discussed as candidate mechanisms influencing circuit formation and disease modification [39,130,139,140]. On the AK-related side, abnormalities in adenosine kinase have been linked to seizures, cognitive impairment, and disrupted synaptic plasticity. However, caution is required before generalising such findings across all rare neurological diseases [141,142].

Research on these disease groups has provided an opportunity to reassess extracellular ATP-dependent purinergic dysregulation at the intersection of neurodevelopment, neuroinflammation, and metabolic homeostasis, although most potential therapeutic applications remain at the preclinical stage [39,130,139,140]. Strategies targeting P2X7 receptors, ectonucleotidase systems, or adenosine-related metabolic enzymes may therefore be promising, but they will require validation that takes into account disease-specific pathology, developmental stage, and cell-type specificity [113,126,140,141].

Recent advances in fluorescent and genetically encoded sensors that track extracellular ATP and adenosine in vivo are beginning to reveal that neurons, astrocytes, and microglia exhibit distinct release and uptake patterns depending on stimulation conditions and disease context [22,143,144,145]. In parallel, microglial responses mediated by P2X7 receptors and the conversion of extracellular ATP to adenosine through ectonucleotidase systems including CD39 and CD73 are now being recognised as regulatory mechanisms that may contribute both to amplification and to resolution of inflammation [113,116,126,140,146]. Future work will need to integrate these extracellular purine metabolic systems with intracellular AK-AMPK-dependent metabolic responses. Such efforts may improve our understanding of disease-specific pathological mechanisms.

To understand the pathological significance of extracellular ATP-dependent purinergic dysregulation described above, it is essential to clarify which cell types release extracellular ATP and adenosine and through which mechanisms these signals are released and converted. In the next section, we therefore shift our focus to ATP and adenosine release mechanisms and intercellular interactions in neurological disease, with particular attention to the respective roles of neurons, astrocytes, and microglia.

## 5. ATP/Adenosine Release Mechanisms and Intercellular Interactions in Neurological Disorders

To understand the pathophysiological significance of extracellular ATP dynamics described in the previous section, it is necessary to clarify which cell types release ATP and adenosine and through which mechanisms these molecules are released and converted. In neurological disorders, these release and degradation pathways are closely linked to intercellular interactions, neural circuit function, and the formation of inflammatory environments. In this section, we therefore summarise ATP and adenosine release mechanisms, with particuar emphasis on neurons, astrocytes, and microglia, and discuss their significance in intercellular communication.

### 5.1. Cellular Mechanisms of ATP/Adenosine Release and Neural Activity

Increases in extracellular ATP and adenosine during neuronal activity influence excitability, inhibition, and neurotransmitter release [22,116,117,119]. Numerous studies have shown that ATP and adenosine release can be triggered by electrical stimulation of nerve fibres or neuronal depolarisation using electrophysiological or optogenetic approaches [139,144,145]. Importantly, the mechanisms of ATP and adenosine release vary across brain regions [22,139,145].

### 5.2. Mechanisms of ATP Release from Neurons

Multiple mechanisms have been proposed to mediate ATP release from neurons [124,128,146,147]. One representative mechanism is activity-dependent exocytosis, in which ATP is thought to be stored in vesicles and released from presynaptic terminals in response to neuronal activity [62,124,127]. Vesicular nucleotide transporter (VNUT) is considered to participate in this process. Evidence from cultured hippocampal neurons and recordings of P2X receptor-dependent postsynaptic currents supports this mechanism, although the contribution of P2X receptor currents is relatively small compared with AMPA/KA receptor-mediated transmission [62,127,128]. In addition, neurons may release ATP through volume-regulated anion channels (VRACs) along axons and through pannexin-1 channels on somata and dendrites [147,148,149]. However, their quantitative contribution under physiological conditions remains incompletely defined.

Evidence for neuronal ATP release has accumulated from both direct and indirect approaches. Direct bioluminescent measurements have demonstrated increases in extracellular ATP after high-frequency stimulation, although methodological artefacts have also been raised as a concern [150,151,152]. Indirect evidence includes recordings of P2X receptor-dependent currents and calcium signals detected in astrocytes in response to neuron-derived ATP release [62,127,152,153,154]. Even so, direct measurements of activity-dependent ATP release in situ or in vivo remain limited [143,144,152]. Thus, technical challenges remain substantial, and further validation is required to exclude stimulation-induced artefacts such as electroporation [150,151,152]. Although several release pathways have been proposed, their respective physiological and pathological contributions remain insufficiently defined.

### 5.3. Mechanisms of ATP Release from Astrocytes

Multiple candidate pathways have been proposed for ATP release from astrocytes, including pannexins, connexin hemichannels, P2X7 receptors, mechanosensitive channels, and VRACs [148,153,155,156,157,158]. Connexin hemichannels can open under low extracellular Ca^2+^ conditions and may mediate ATP release [154,155,156]. Recent development of fluorescent sensors such as GRABATP1.0 has enabled direct measurement of extracellular ATP dynamics, allowing local events to be distinguished from propagating waves and making it possible to assess spatiotemporal release patterns in greater detail [22,144,153]. More recently, adenosine itself has been shown to regulate astrocyte metabolism and support neuronal activity, supporting the view that astrocytes should be regarded not simply as ATP sources but as active metabolic hubs that sense adenosine and regulate brain metabolism [159,160]. Activity-dependent ATP waves and focal ATP release have also been observed in cortical tissue [144,153,154]. However, the precise molecular mechanisms underlying these events remain incompletely defined, and targeted genetic validation will be needed in future studies [153,156,161].

Mechanosensitive channels and hemichannels have also been suggested to participate in astrocytic ATP release and synaptic plasticity [155,157,158]. With regard to Ca^2+^-dependent release, astrocytes are thought to respond to neurotransmitter stimulation through Ca^2+^-mediated ATP release, and exocytosis involving VNUT, lysosomal compartments, and activity-linked vesicular mechanisms is considered one of the major candidate pathways [151,161,162].

### 5.4. Contribution of Microglia to ATP Dynamics

Microglia can also release ATP, although their contribution to extracellular ATP dynamics has received less attention than that of neurons or astrocytes. Imura and colleagues reported that microglia release ATP through exocytosis [163]. In cultured microglia, ATP-related responses are known to be enhanced by inflammatory stimulation, suggesting a close link to inflammatory status [121,128,139]. In addition, recent in vivo sensor studies have shown that local cortical ATP events increase after inflammatory stimulation, further drawing attention to the relationship between ATP dynamics and microglial responses [139,149,163].

Microglia also possess channel-mediated ATP release pathways. Chu and colleagues demonstrated that SWELL1, a VRAC component, contributes to ATP release from microglia and is involved in pain-like behaviour in a chronic compression model of neuropathic pain [164]. In addition, microglia sense extracellular ATP/ADP gradients through P2Y12 receptors and extend their processes towards sites of neuronal activity, thereby participating in local ATP dynamics and neuroprotective responses [165,166].

### 5.5. Adenosine Release and Conversion Mechanisms in the Brain

Neurons may directly release adenosine through activity-dependent mechanisms, and the released adenosine is thought to influence short-term plasticity via A1 receptors [122,152,154,167]. Equilibrative nucleoside transporters (ENTs) are thought to participate in this release pathway. Neuronal activity can induce synaptic suppression through A1 receptor activation, and this effect may be attenuated by ENT inhibition [122,152,167]. This process may involve Na^+^ influx during activity and the consequent increase in ATP consumption by Na^+^/K^+^-ATPase, and the involvement of L-type voltage-dependent Ca^2+^ channels has also been suggested. At the same time, ATP released from astrocytes can be converted by CD39/CD73 into extracellular adenosine, thereby providing an additional source of adenosine in the brain [22,125,126,151]. This conversion pathway may act through presynaptic A1 receptors to suppress transmission or through A2A receptors to facilitate it [120,122,124]. Thus, neurons and astrocytes may contribute to the extracellular adenosine pool through distinct mechanisms—direct transport and ATP-to-adenosine conversion, respectively. However, the full spatiotemporal dynamics of this system remain incompletely resolved.

Astrocyte-derived ATP is considered one of the major extracellular sources of adenosine because it can be converted by the ectonucleotidases CD39 and CD73 [22,125,126,151]. In some systems, astrocytic ATP release can be induced by activation of non-NMDA receptors and mGluR5 [159,168,169]. The resulting adenosine may then act on presynaptic A1 receptors to mediate heterosynaptic inhibition or on A2A receptors to enhance glutamate release [120,122,124]. Studies using astrocyte manipulation and mice with impaired vesicular release support an important role for this pathway in synaptic regulation [159,168,170]. Although this framework has become increasingly well supported, the complete spatiotemporal dynamics of ATP and adenosine signalling remain unresolved.

Microglia may also participate in regulation of adenosine levels in the brain and thereby influence neuronal activity [125,139,150,151]. These cells express purinergic receptors, and P2Y12 receptors in particular are thought to be important for sensing extracellular ATP released from neurons and astrocytes during tissue damage and subsequent neuronal activity [151,165,166]. Upon sensing ATP, microglia extend their processes towards the source of release and are thought to form neuroprotective interactions with neurons [165,166,171]. P2Y12 receptor deficiency can exacerbate seizures and injury after cerebral artery occlusion, suggesting functional interplay with adenosine-mediated neuroprotection [151,165,166]. In at least some reports, microglia express CD39, which converts ATP to AMP, whereas neurons express CD73, which converts AMP to adenosine [125,126,151]. This two-step process may contribute to suppression of neuronal hyperactivity through activation of neuronal A1 receptors. Indeed, microglial depletion or CD39 knockout has been associated with reduced adenosine levels, increased neuronal excitability, and worsening of chemically induced seizures [126,151,165]. More recently, microglia-derived CD39 has also been implicated in maintenance of basal cerebral blood flow and neurovascular coupling, supporting the idea that CD39 is not merely a degradative enzyme but a key mediator linking ATP-to-adenosine conversion with neural activity and vascular responses [125,151].

Taken together, the release and conversion mechanisms of ATP and adenosine, together with glia–neuron interactions, provide a fundamental basis for controlling circuit excitability and metabolic homeostasis. In the next section, we examine how these molecular and cellular mechanisms become disrupted in representative neurological disorders such as Alzheimer’s disease, Parkinson’s disease, and epilepsy, and how such disruption contributes to disease phenotypes.

## 6. Metabolic Abnormalities in Neurodegenerative and Hyperexcitable Disorders

The mechanisms discussed in the previous sections become clinically relevant in representative neurological diseases as distinct patterns of mitochondrial dysfunction, glia-dependent inflammation, and neural circuit instability. Although Alzheimer’s disease, Parkinson’s disease, and epilepsy differ markedly in phenotype, all can be interpreted within a shared framework of disrupted energy homeostasis, impaired intercellular metabolic coordination, and purinergic dysregulation.

At the same time, the role of metabolism is not identical across these disorders. Alzheimer’s disease is characterised by a relatively chronic and prodromal decline in cerebral metabolism, Parkinson’s disease by selective vulnerability of highly mitochondria-dependent dopaminergic neurons, and epilepsy by acute surges in energy demand during seizures, together with maladaptive interictal reprogramming. Comparison of these disorders therefore requires attention to differences in time scale, vulnerable cell populations, and the dominant metabolic bottleneck through which circuit failure emerges.

**Figure 4:**
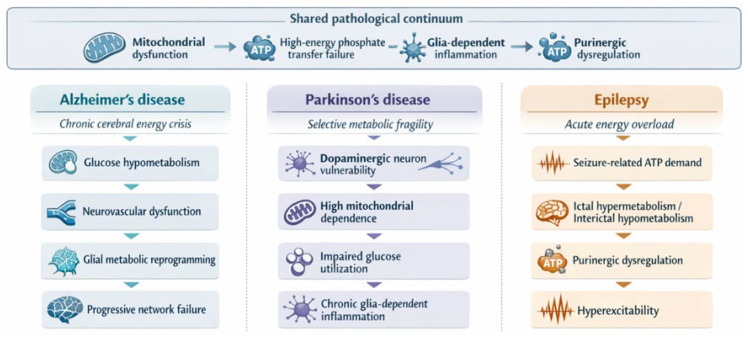
Shared and disease-specific metabolic abnormalities in Alzheimer’s disease, Parkinson’s disease, and epilepsy. This figure compares Alzheimer’s disease, Parkinson’s disease, and epilepsy within a shared pathological continuum while highlighting their disease-specific modes of metabolic expression. Across all three disorders, mitochondrial dysfunction, failure of high-energy phosphate transfer, glia-dependent inflammation, and purinergic dysregulation are presented as shared elements of disrupted energy homeostasis. Within this common framework, Alzheimer’s disease is characterised primarily by a chronic cerebral energy crisis involving glucose hypometabolism, neurovascular dysfunction, glial metabolic reprogramming, and progressive network failure. Parkinson’s disease is depicted as a disorder of selective metabolic fragility, in which vulnerability of dopaminergic neurons is shaped by high mitochondrial dependence, impaired glucose utilisation, and chronic glia-dependent inflammation. Epilepsy is represented as a disorder of acute energy overload, in which seizure-related ATP demand, ictal hypermetabolism with interictal hypometabolism, purinergic dysregulation, and circuit hyperexcitability destabilise neuronal function. The comparative footer further highlights differences in disease time scale, the primary site of vulnerability, and the dominant metabolic bottleneck, thereby illustrating how shared mechanisms generate disease-specific patterns of circuit dysfunction across neurological disorders. Created in BioRender. Tao, H. (2026) https://BioRender.com/wkbvbl9, accessed on 10 June 2026.

The following sections compare these representative disorders from a common perspective—how mitochondrial dysfunction, failure of high-energy phosphate transfer, glia-dependent inflammation, and purinergic dysregulation converge to impair circuit stability—while tracing their shared and disease-specific modes of metabolic expression. This comparative structure is summarised in Figure 4 and Table 1. In this way, Section 6.1, Section 6.2 and Section 6.3 move from chronic cerebral energy crisis to selective metabolic fragility to acute energy overload, thereby clarifying both shared therapeutic opportunities and the need for disorder-specific interpretation.

### 6.1. Alzheimer’s Disease: Progressive Cerebral Energy Crisis

Alzheimer’s disease provides a representative example of a progressive cerebral energy crisis. Because the brain has an exceptionally high energy demand, it is particularly vulnerable to disturbances in energy supply and mitochondrial function. In Alzheimer’s disease, increasing evidence suggests that abnormalities in energy metabolism are closely linked to neuronal dysfunction, neurodegenerative progression, and cognitive decline [5,8,26,30]. In addition to extracellular β-amyloid (Aβ) deposition and intracellular neurofibrillary changes, Alzheimer’s disease is characterised by cerebrovascular alterations, blood–brain barrier disruption, and neuroinflammation, all of which may collectively contribute to neuronal loss [8,25,178,179].

Mitochondrial dysfunction is thought to play a central role in the progression of Alzheimer’s disease. Under physiological conditions, neurons, astrocytes, microglia, oligodendrocytes, and the neurovascular unit function as an integrated metabolic network, but in Alzheimer’s disease, this coordination becomes progressively disrupted [31,33,34,179,180]. Reduced neuronal glucose uptake, impaired insulin signalling, decreased endothelial GLUT1 expression at the blood–brain barrier, and altered astrocytic and microglial metabolic states together contribute to a vulnerable cerebral energy environment [25,35,36,172,178,179,180,181,182,183,184,185].

A representative example of this vulnerability is the characteristic 18F-FDG PET pattern in Alzheimer’s disease, in which hypometabolism is most evident in the posterior cingulate cortex, precuneus, and temporoparietal association cortex, often already at the prodromal or mild cognitive impairment stage. This regional pattern indicates that activity-dependent glucose utilisation becomes impaired before widespread neuronal loss is apparent and provides a measurable link between neurovascular dysfunction, glial metabolic disturbance, and neuronal energy failure [26,30,178,179,180,181,182,183,184,185].

The relationship between Aβ, tau, and cerebral hypometabolism further illustrates this process. Soluble Aβ species are thought to impair synaptic transmission, mitochondrial respiration, and neuronal glucose utilisation early in the disease, thereby contributing to the initial hypometabolic state. As pathology progresses, tau hyperphosphorylation and aggregation become more closely associated with regional network dysfunction, cortical atrophy, and cognitive decline. Thus, Aβ may act mainly as an upstream trigger of metabolic stress, whereas tau more closely reflects the anatomical spread and clinical severity of neuronal failure [181,182,183,186,187,188].

Overall, Alzheimer’s disease can be viewed as a disorder in which neurovascular dysfunction, glial metabolic reprogramming, and proteinopathy-driven neuronal stress converge to produce a progressive cerebral energy crisis. This perspective also supports interest in metabolism-based interventions, including support of glucose transport, restoration of insulin-related signalling, and correction of glia–neuron metabolic coupling, although the therapeutic rationale remains context-dependent [5,8,25,172,178,179,185,186].

### 6.2. Parkinson’s Disease: Selective Neuronal Metabolic Fragility

Parkinson’s disease provides a representative example of selective neuronal metabolic fragility. It is a progressive neurodegenerative disorder characterised by degeneration of dopaminergic neurons in the substantia nigra and dysfunction of the nigrostriatal pathway [173,174,189]. These neurons are especially vulnerable because of their high mitochondrial dependence, sustained pacemaking activity, oxidative burden related to dopamine metabolism, and age-associated decline in metabolic resilience [45,46,174,190]. Parkinson’s disease should therefore be viewed not only as a proteinopathy or neurotransmitter disorder but also as a condition of selective metabolic fragility.

This fragility is reflected in impaired glucose metabolism and altered substrate utilisation in both neurons and glia [172,176,191]. Although glycolysis can provide rapid local ATP supply, its compensatory capacity may become insufficient in the setting of mitochondrial dysfunction, reduced glucose handling, and broader metabolic reprogramming [15,172,176,192]. As a result, dopaminergic neurons may fail to meet energetic demand, while astrocytes and microglia undergo metabolic shifts that further reshape the local tissue environment [172,176,189,193].

Glia-dependent inflammation further amplifies this process. Activated microglia in Parkinson’s disease tend to shift towards glycolysis and pro-inflammatory signalling, thereby increasing reactive oxygen species production, NF-κB-related inflammatory activity, and secondary stress on dopaminergic neurons [47,189,193,194]. In this way, mitochondrial dysfunction, abnormal glucose metabolism, and glia-dependent inflammation form a self-reinforcing cycle that helps sustain disease progression.

The MPTP model provides a particularly clear illustration of how mitochondrial energy failure can lead to selective dopaminergic neurodegeneration. After systemic exposure, MPTP is converted to its active metabolite MPP+, which is preferentially taken up into dopaminergic neurons through the dopamine transporter and accumulates within mitochondria. There, MPP+ inhibits mitochondrial complex I, resulting in impaired oxidative phosphorylation, ATP depletion, increased reactive oxygen species production, and activation of cell death pathways. This mechanism provides an experimental framework for understanding how transporter-dependent toxin accumulation and mitochondrial respiratory failure can converge to produce selective loss of substantia nigra dopaminergic neurons in Parkinsonian pathology [195,196,197,198].

Taken together, Parkinson’s disease can be viewed as a disorder in which selective neuronal vulnerability emerges from the convergence of mitochondrial dysfunction, impaired glucose utilisation, and chronic glia-dependent inflammation. This perspective also supports interest in metabolism-based interventions, including correction of glucose transporter expression, support of substrate utilisation, and enhancement of glycolytic compensation, although the therapeutic rationale remains context-dependent [176,177,191,193,194].

### 6.3. Epilepsy: Acute Energy Overload and Purinergic Dysregulation

Epilepsy provides a representative example of acute energy overload with purinergic dysregulation. It differs from neurodegenerative disorders in that its metabolic disturbance is dominated by acute surges in energy demand during seizures, together with maladaptive reprogramming during interictal periods [3,68,146,175,199]. Because repetitive neuronal firing imposes an abrupt ATP burden on ion pumps, synaptic vesicle cycling, and neurotransmitter clearance, seizure activity provides a particularly clear example of how failure of energy homeostasis can destabilise neural circuits.

This vulnerability is reflected in the bidirectional relationship between seizures and metabolism. During acute seizures, glucose consumption rises sharply, glycolytic flux is enhanced, and more pyruvate is diverted to lactate production rather than complete oxidation through the TCA cycle. This shift can provide rapid local ATP but at lower energetic efficiency, helping to explain the characteristic contrast between ictal hypermetabolism and interictal hypometabolism [21,29,146,175,192,199]. In parallel, restricted mitochondrial flux and impaired oxidative metabolism may further limit the ability of neurons to sustain prolonged activity [3,68,199].

A representative example can be considered in hippocampal circuits during prolonged high-frequency discharge. As excitatory neurons fire repetitively, ATP demand rises abruptly because Na^+^/K^+^-ATPase activity and synaptic vesicle cycling must be continuously maintained. Extracellular adenosine may transiently increase and act through A1 receptors to dampen excitability, but if seizure activity is sustained, this endogenous brake can become insufficient. At the same time, ATP release from neurons and glia, including release through pannexin-1-related pathways, together with P2X7-associated inflammatory signalling, may further destabilise neuron–glia communication and aggravate local energy failure [124,146,147,160,200,201,202,203].

A further example is provided by chronic changes in adenine nucleotide regulation and adenosine clearance. AK5, a brain-enriched AK isoform, is downregulated in temporal lobe epilepsy tissue and experimental models, suggesting impaired intracellular adenine nucleotide buffering under chronic hyperexcitability [96]. In parallel, increased astrocytic adenosine kinase expression is thought to lower extracellular adenosine availability and weaken the endogenous A1 receptor-mediated anticonvulsant tone [159,160]. Thus, epilepsy illustrates how both loss of intracellular nucleotide stabilisation and reduced adenosine-dependent inhibition can shift circuits toward persistent hyperexcitability.

Taken together, epilepsy may be understood as a disorder in which acute energy overload, impaired mitochondrial support, and purinergic dysregulation converge to destabilise neural circuits and maintain hyperexcitability. This perspective supports further interest in metabolism-based interventions, including modulation of adenosine metabolism, inhibition of P2X7-related inflammatory pathways, and strategies to improve substrate utilisation, although their therapeutic rationale remains context-dependent [124,146,147,148,175,192,200].

Together, these disorders can be interpreted as disease-specific expressions of a shared pathological continuum involving mitochondrial dysfunction, failure of high-energy phosphate transfer, glia-dependent inflammation, and purinergic dysregulation. This perspective leads naturally to the next section, which considers shared therapeutic opportunities while preserving disorder-specific interpretation.

## 7. Therapeutic Opportunities and Future Perspectives

The comparative framework developed in the previous section suggests that metabolism-based interventions may offer shared therapeutic opportunities across neurological disorders because several disease processes converge on partially overlapping axes of vulnerability, including mitochondrial dysfunction, failure of high-energy phosphate transfer, glia-dependent inflammation, and purinergic dysregulation. Importantly, these mechanisms are not expressed identically across diseases: in Alzheimer’s disease, they unfold as a relatively chronic cerebral energy crisis; in Parkinson’s disease, as selective metabolic fragility of highly mitochondria-dependent dopaminergic neurons; and in epilepsy, as recurrent acute energy overload with maladaptive purinergic regulation. A common therapeutic logic can therefore be considered across disorders, but its timing, cellular targets, and expected effects must still be interpreted in a disease-specific manner. In the following subsections, we consider cross-disease therapeutic opportunities related to mitochondrial support and high-energy phosphate transfer, integrative disease interpretation, AK-targeted intervention, and the translational potential of the eATP–AK axis, while emphasising constraints imposed by disease stage, vulnerable cell populations, and circuit context.

### 7.1. Mitochondrial Support and High-Energy Phosphate Transfer

Mitochondrial support and preservation of high-energy phosphate transfer lie upstream of the energy supply–demand imbalance observed across multiple neurological disorders and therefore remain plausible cross-disease therapeutic strategies. However, the therapeutic rationale differs by disease context: in Alzheimer’s disease, such strategies may aim to slow a chronic cerebral energy crisis; in Parkinson’s disease, to preserve highly mitochondria-dependent dopaminergic neurons; and in epilepsy, to improve resilience against recurrent acute energy overload. Interventions directed at these processes may help preserve local ATP availability and secondarily reduce oxidative stress, synaptic dysfunction, and activation of cell death pathways. This perspective also highlights the need for careful attention to timing and cellular specificity when applying such strategies across disorders.

### 7.2. Integrative Interpretation and Translational Limits

An integrative interpretation is necessary to explain the persistence and heterogeneity of progressive pathology in neurological disorders, but this perspective also reveals important translational limits. The comparison of Alzheimer’s disease, Parkinson’s disease, and epilepsy indicates that metabolic abnormalities, inflammation, vascular dysfunction, and proteostatic failure should be viewed as interconnected elements of a broader pathological network. This integrative perspective is essential not only for disease interpretation but also for designing interventions that target shared mechanisms without obscuring clinically important differences in disease expression.

Because these pathological domains can mutually amplify one another, therapies centred on a single target may be insufficient to modify the overall disease process. This point is especially relevant when translating metabolism-based interventions across disorders: a pathway that appears protective in one disease stage or cell type may be ineffective or even maladaptive in another. The comparative framework presented here therefore supports combined consideration of biomarker design, disease-stage stratification, and cell-type specificity.

At the same time, the integrated model proposed in this review should still be regarded as a working hypothesis rather than a finalised causal map. Although the framework linking mitochondrial dysfunction, the CK/PCr system, the AK-AMPK system, and extracellular ATP-dependent purinergic dysregulation is supported in part by established evidence, several links remain incompletely validated in human brain disease. Moreover, many of the cited data derive from cellular or animal models, and oversimplified unification across Alzheimer’s disease, Parkinson’s disease, and epilepsy should be avoided. This perspective also highlights the need for comparative validation across diseases, stages, and cellular contexts.

### 7.3. AK-Targeted Intervention and Research Priorities

AK-targeted intervention is of particular translational interest because the AK system connects adenine nucleotide buffering to metabolic signalling, stress adaptation, and potentially the extracellular purine environment. At the same time, its therapeutic relevance is unlikely to be uniform across disorders: AK-related strategies may be more informative in conditions where adenine nucleotide instability, AMPK-linked maladaptation, or purinergic imbalance are especially prominent. AK-targeted intervention may therefore be best considered within a comparative framework that takes into account isoform specificity, cellular localisation, and disease context.

Future work will need to define how individual AK isoforms are regulated across neuronal and glial populations; how they interact with glycolysis, oxidative phosphorylation, and nucleotide salvage pathways; and whether their modulation can improve recovery after metabolic stress. It will also be important to test the efficacy and safety of AK-targeted interventions in models that capture disease-specific vulnerability, including neurodegenerative, hyperexcitable, and ischemic conditions [16,17,90,96]. This perspective also highlights the need for disease-specific experimental models when evaluating AK-targeted strategies.

### 7.4. The eATP–AK Axis: Opportunities and Constraints

The eATP–AK axis offers therapeutic opportunities, but it also poses important translational constraints because it could, in principle, modulate both extracellular inflammatory signalling and intracellular metabolic regulation. This duality is particularly relevant in disorders such as epilepsy and Parkinson’s disease, where purinergic dysregulation and glia-dependent inflammation appear closely linked to circuit instability, but it may also have broader implications for chronic neurodegenerative conditions. At the same time, translation of this strategy requires careful alignment of receptor subtype specificity, cell-type selectivity, blood–brain barrier penetration, and disease stage.

Several translational constraints remain. Receptor subtype diversity and off-target effects must be controlled, excessive disturbance of intracellular nucleotide balance should be avoided, and biomarkers capable of reflecting extracellular ATP dynamics, AK-related metabolic state, and downstream purinergic dysregulation will be required to guide patient selection and response evaluation. These requirements underscore that combined modulation of purinergic dysregulation and energy metabolism is conceptually promising but technically demanding.

Future efforts should therefore integrate small-molecule, gene-based, and combination strategies with biomarker-guided evaluation in order to determine when targeting the eATP–AK axis is most relevant and in which disease contexts it is most likely to be beneficial. This translational agenda aligns with the broader conclusion of this review: shared metabolic mechanisms may reveal common therapeutic opportunities, but effective application will depend on disorder-specific interpretation and biomarker-guided translation.

## 8. Conclusions

Taken together, disruption of energy homeostasis in neurological disorders may be reinterpreted as a shared pathological continuum linking mitochondrial dysfunction, failure of high-energy phosphate transfer, glia-dependent inflammation, and extracellular ATP-dependent purinergic dysregulation. Comparison of Alzheimer’s disease, Parkinson’s disease, and epilepsy further suggests that this framework captures shared and disease-specific manifestations of disrupted energy homeostasis: chronic cerebral energy crisis in Alzheimer’s disease, selective metabolic fragility in Parkinson’s disease, and acute energy overload with purinergic dysregulation in epilepsy. Energy homeostasis disruption may therefore be viewed not as a single lesion but as a multi-level disturbance that emerges differently according to disease time scale, vulnerable cell populations, and circuit context. From this perspective, the neurological disorders discussed in this review may also be interpreted within a broader conceptual framework in which apparently distinct pathological states are understood as diverse expressions of a common underlying principle. This view preserves disease-specific differences while situating them within a shared pathological foundation, thereby emphasising both heterogeneity and unity within the proposed continuum.

From a therapeutic perspective, this comparative view helps explain why metabolism-based interventions may reveal shared therapeutic opportunities across neurological disorders while cautioning against oversimplified unification. Approaches aimed at supporting mitochondrial function, improving glucose or substrate utilisation, correcting glia–neuron metabolic coupling, or modulating extracellular ATP/adenosine dynamics may address common axes of vulnerability, but their relevance is likely to depend on disease stage, cellular context, and disorder-specific interpretation. In this respect, the finding that secretion of cytosolic AK1 is required for extracellular ATP synthesis in differentiated C2C12 myotubes remains conceptually important because it raises the possibility that AK may influence not only intracellular adenylate homeostasis but also the extracellular purine environment under certain conditions, although such a mechanism has not yet been established in the nervous system [115].

Looking ahead, future research should proceed in at least three directions. First, the comparative framework proposed here should be tested more rigorously across diseases to determine which components of metabolic vulnerability belong to a shared pathological continuum and which require disorder-specific interpretation. Second, single-cell analysis, spatial omics, metabolic flux analysis, live imaging, and patient-derived iPS cell models should be integrated to define when and where metabolic vulnerability emerges, including the cell-type- and compartment-specific roles of AK isoforms, their complementarity with the CK system, and their relationships to extracellular ATP dynamics, purinergic dysregulation, and glia-dependent inflammation. Third, clinically relevant biomarkers—such as 31P-MRS-based indices, extracellular purine metabolic markers, and dynamic indicators of substrate utilisation—will be needed to translate this framework into disease stratification and personalised therapeutic intervention.

## Figures and Tables

**Table 1:** Comparative summary of mitochondrial dysfunction, related disturbances, and therapeutic opportunities in Alzheimer’s disease, Parkinson’s disease, and epilepsy.

Category	Alzheimer’s Disease	Parkinson’s Disease	Epilepsy
Dominant metabolic phenotype	Chronic cerebral energy crisis, with progressive glucose hypometabolism and network failure [25,30,172].	Selective metabolic fragility of dopaminergic neurons in the nigrostriatal system [45,173,174].	Acute energy overload during seizures, followed by maladaptive interictal metabolic reprogramming [68,146,175].
Major mitochondrial dysfunction	Reduced oxidative phosphorylation; impaired mitochondrial dynamics and quality control; increased ROS generation; calcium dysregulation; interaction with Aβ- and tau-associated stress [5,8,45].	Complex I-related respiratory impairment; defective mitophagy and mitochondrial transport; oxidative stress linked to dopamine metabolism; calcium burden from pacemaking activity; α-synuclein-associated mitochondrial stress [45,46,47].	Failure to match seizure-related ATP demand; restricted oxidative metabolism; increased ROS production; calcium overload; mitochondrial injury during recurrent hyperexcitation; reduced metabolic reserve [3,68,175].
Related disturbances	Neurovascular dysfunction; reduced glucose transport and insulin-related signalling; glial metabolic reprogramming; CK/PCr vulnerability; glia-dependent inflammation; purinergic imbalance [25,31,49].	Impaired glucose utilisation; insufficient glycolytic compensation; chronic microglial activation; NF-κB-related inflammatory signalling; oxidative-inflammatory amplification; possible high-energy phosphate transfer vulnerability [47,172,176].	Ictal hypermetabolism with interictal hypometabolism; lactate accumulation; impaired CK/PCr compensation; AK5 downregulation; adenosine depletion through increased adenosine kinase activity; P2X7-related inflammation; circuit hyperexcitability [96,124,141,146].
Therapeutic opportunities	Support mitochondrial resilience; improve glucose/substrate utilisation; restore glia–neuron metabolic coupling; modulate inflammation and extracellular ATP/adenosine balance; enable biomarker-guided early intervention [5,25,172].	Enhance mitochondrial quality control; reduce oxidative and inflammatory stress; support glucose transporter expression and substrate utilisation; preserve dopaminergic bioenergetics; apply context-dependent purinergic modulation [45,176,177].	Strengthen metabolic resilience; use ketogenic or substrate-based strategies where appropriate; modulate adenosine metabolism; inhibit maladaptive P2X7-related signalling; support nucleotide buffering; tailor intervention to seizure stage [124,141,175].

## Data Availability

No new data were created or analysed in this study. Data sharing is not applicable to this article.

## Abbreviations

The following abbreviations are used in this manuscript:

CK	creatine kinase
AK	adenylate kinase
CK/PCr	creatine kinase/phosphocreatine
AK-AMPK	adenylate kinase/AMP-activated protein kinase
TCA	tricarboxylic acid
ROS	reactive oxygen species
ANLS	astrocyte–neuron lactate shuttle
VNUT	vesicular nucleotide transporter
VRACs	volume-regulated anion channels
